# Fast Periodic Auditory Stimulation Reveals a Robust Categorical Response to Voices in the Human Brain

**DOI:** 10.1523/ENEURO.0471-20.2021

**Published:** 2021-06-17

**Authors:** Francesca M. Barbero, Roberta P. Calce, Siddharth Talwar, Bruno Rossion, Olivier Collignon

**Affiliations:** 1 Institute of Research in Psychology and Institute of Neuroscience, Louvain Bionics Center, University of Louvain, Louvain-la-Neuve 1348, Belgium; 2 Université de Lorraine, Centre National de la Recherche Scientifique, Centre de Recherche en Automatique de Nancy, Nancy F-54000, France; 3 Université de Lorraine, Service de Neurologie, Centre Hospitalier Régional et Universitaire-Nancy F-54000, France; 4 Center for Mind/Brain Sciences, University of Trento, Rovereto 38068, Italy

**Keywords:** auditory cortex, electroencephalography, frequency tagging, voice

## Abstract

Voices are arguably among the most relevant sounds in humans’ everyday life, and several studies have suggested the existence of voice-selective regions in the human brain. Despite two decades of research, defining the human brain regions supporting voice recognition remains challenging. Moreover, whether neural selectivity to voices is merely driven by acoustic properties specific to human voices (e.g., spectrogram, harmonicity), or whether it also reflects a higher-level categorization response is still under debate. Here, we objectively measured rapid automatic categorization responses to human voices with fast periodic auditory stimulation (FPAS) combined with electroencephalography (EEG). Participants were tested with stimulation sequences containing heterogeneous non-vocal sounds from different categories presented at 4 Hz (i.e., four stimuli/s), with vocal sounds appearing every three stimuli (1.333 Hz). A few minutes of stimulation are sufficient to elicit robust 1.333 Hz voice-selective focal brain responses over superior temporal regions of individual participants. This response is virtually absent for sequences using frequency-scrambled sounds, but is clearly observed when voices are presented among sounds from musical instruments matched for pitch and harmonicity-to-noise ratio (HNR). Overall, our FPAS paradigm demonstrates that the human brain seamlessly categorizes human voices when compared with other sounds including musical instruments’ sounds matched for low level acoustic features and that voice-selective responses are at least partially independent from low-level acoustic features, making it a powerful and versatile tool to understand human auditory categorization in general.

## Significance Statement

Voices are arguably among the most relevant sounds we hear in our everyday life, and several studies have corroborated the existence of regions in the human brain that respond preferentially to voices. However, whether this preference is driven by specific acoustic properties of voices or whether it rather reflects a higher-level categorization response to voices is still under debate. We propose a new approach to objectively identify rapid automatic voice-selective responses with frequency tagging and electroencephalographic (EEG) recordings. In 4 min of recording only, we recorded robust voice-selective responses independent from low-level acoustic cues, making this approach highly promising for studying auditory perception in children and clinical populations.

## Introduction

Voices of conspecifics are arguably among the most relevant sounds we hear in our everyday life: they do not only carry speech, but also convey a wealth of information about the speakers such as their sex, age, emotional status, identity, trustworthiness, etc. (Belin et al., 2004). Regions in the human brain located along the bilateral superior temporal sulcus (STS) respond more to human voices than to other sounds (temporal voice areas; TVAs), playing a key role in voice recognition (Belin et al., 2000, 2002). However, whether this selectivity is fully accounted for by specific acoustic properties of voices (Staeren et al., 2009; Moerel et al., 2012; Ogg et al., 2020), or whether it also reflects a higher-level categorization response beyond these low-level auditory properties is still under debate. This question has been previously addressed through careful choice and design of acoustic stimuli (Belin et al., 2002; Levy et al., 2003; Agus et al., 2017) and sophisticated signal analyses (Leaver and Rauschecker, 2010; Moerel et al., 2012; Giordano et al., 2013), with results sometimes challenging the notion of brain regions dedicated to the abstract encoding of voices (Santoro et al., 2017; Ogg et al., 2020). For instance, regions identified as voice-selective present a response bias to low frequencies typical of voices, even when responding to tones (Moerel et al., 2012), and portions of auditory cortex are sensitive to the degree of harmonic structure present in both artificial sounds and in human vocalizations (Lewis et al., 2009).

The approaches used so far to delineate voice-selectivity in the human brain, mostly relying on functional magnetic resonance imaging (fMRI) or event-related potential recordings with electroencephalography (EEG), present limitations that hinder the characterization of a putative high-level voice-categorization response. For instance, these methods usually imply the subtraction between neural responses elicited by voices and control sounds which occurred at different times, or the regression of parameters linked to low-level properties of sounds, when in fact the subtracted components might be a part of the expression of a response to voices (Frühholz and Belin, 2018).

Here, we shed light on the nature of voice-selective responses in the human brain by proposing a new approach to identify these automatic responses objectively and directly (i.e., without subtraction/regression). This approach relies on EEG recordings and, more specifically, on “EEG frequency tagging” (Regan, 1989). Frequency tagging builds on the principles of so-called steady-state evoked responses: under periodic external stimulation, the brain region encoding that input responds at the exact same stimulation frequency (for review, see Norcia et al., 2015). We developed a fast periodic auditory stimulation (FPAS) paradigm adapted from studies in vision, in particular to study face (Rossion et al., 2015; Retter and Rossion, 2016) and letter/word categorization (Lochy et al., 2015). Specifically, participants listened to sequences of heterogeneous sounds presented at a periodic rate of 4 Hz. Critically, each third sound presented in the sequences was a (different) human voice excerpt, so that voices were presented at a periodic rate of 1.333 Hz. In the EEG frequency domain, a response at the sound presentation frequency would reflect shared processes between all sounds, while a putative activity at the voice presentation rate would emerge only if the participant’s brain successfully discriminates human voices from other sounds and generalizes across all the diverse vocal samples presented (to maintain periodicity). To further assess whether low-level properties alone could elicit voice-selective responses, we included a second stimulation sequence with identical periodicity constraints using the same sounds but frequency-bins scrambled (Dormal et al., 2018) to preserve the overall frequency content of the original sounds while disrupting their harmonicity and intelligibility. In a second experiment, we implemented a sequence presenting voices among musical instrument notes that were matched for pitch, harmonicity-to-noise ratio (HNR) and spectral center of gravity to control for frequency content of the sounds, harmonicity and within category homogeneity (Belin et al., 2002, 2011).

In summary, the goal of the present study was both conceptual and methodological. We aimed to develop a FPAS paradigm combined with EEG to test whether and to which extent voice-selective responses are partially independent from low-level acoustic features. Since FPAS provides a marker for categorization that is objective, direct and does not require overt responses to voices from the participants, we expect our observations to hold significant value for further characterization of the nature of voice-selectivity in the human brain.

## Materials and Methods

### Experiment 1 - voice versus object sounds

#### Participants

EEG was recorded in 20 participants (age range 19–26 years, 10 female) in experiment 1. Data from four participants were excluded because of the presence of EEG artefacts. All participants were right-handed and reported normal or corrected to normal vision, normal hearing and no history of psychiatric or neurologic disorders. The experiment was approved by the local ethical committee of the University of Louvain (project 2016-25), all participants provided written informed consent and received financial compensation for their participation.

#### Stimuli

**Figure 1.**
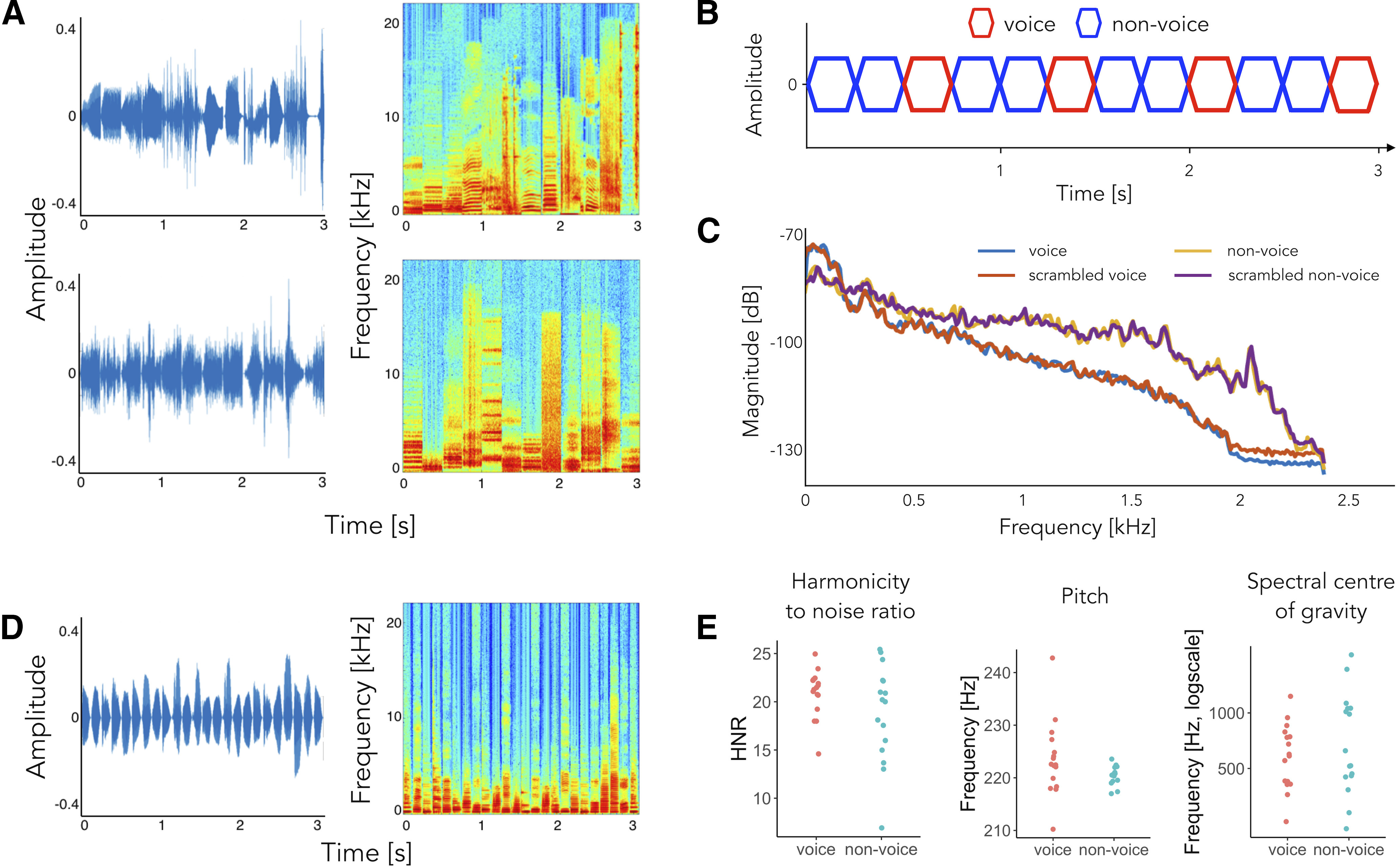
Experimental design for experiments 1 (***A*–*C***) and 2 (***D***, ***E***). ***A***, Three seconds excerpts of the sequences for the *standard* (top) and *scrambled* (bottom) sequences (left) with their relative spectrograms (right). ***B***, Schematic representation of the paradigm. ***C***, *Standard* and *scrambled* sequences: Bode magnitude plot expressing the magnitude in decibels as a function of frequency for the averaged sounds of vocal and non-vocal stimuli separately, for standard and scrambled sequences. ***D***, Three seconds excerpts of the *harmonic* sequence (left) with its spectrogram (right). ***E***, Acoustic features as a function of sound category: HNR, pitch, and spectral center of gravity.

Individual sounds used to create the *standard* sequences were 250 ms long, leading to a base stimulation frequency (BF) of 4 Hz (1/250 ms) and a target frequency (TF) of 1.333 Hz (4 Hz/3: vocal stimuli presented each third sound) and were selected in an effort to be as heterogeneous and variable as possible. We selected 137 non-vocal stimuli including environmental sounds (e.g., water pouring, rain), musical instruments, sounds produced by manipulable and non-manipulable objects (e.g., telephone ringing, ambulance siren). We selected 55 vocal stimuli including speech and non-speech vocalizations pronounced by speakers of different sex, age and emotional states. Stimuli were extracted from various sources including online databases, extracts from audiobooks and the Montreal Affective Voices dataset (Belin et al., 2008). These stimuli were then frequency scrambled using the method described in Dormal et al. (2018) to create the *scrambled* sequences. Specifically, we applied a fast Fourier transformation to vocal and non-vocal sounds and created frequency bins of 200 Hz. Within each of these frequency windows, we shuffled the magnitude and phase of each Fourier component. We then performed an inverse Fourier transform to the signal and then applied the original sound envelope to the scrambled sound. As a result, the scrambled sounds have frequency content and spectral-temporal structure that are almost identical to that of the original stimuli (Fig. 1*C*). However, the harmonicity is altered and the intelligibility of the stimuli is disrupted as confirmed by the behavioral experiment (experiment 3; Fig. 5). All sounds were equalized in overall energy (RMS) and faded-in and faded-out with 10-ms ramps to facilitate individual sounds segregation and avoid clicking.

#### Procedure

A schematic representation of the experimental design is shown in Figure 1*B*. Sounds of the same duration were presented one after another to create periodic auditory sequences. In particular, sounds were presented such that each third sound was a human voice. Vocal and non-vocal samples were selected from various sources and were as heterogeneous as possible to represent the variability characteristic of a sound category (and thus also naturally increasing the variability of low-level acoustic features). No auditory category other than voice was presented periodically. Participants listened to two different sequence types that were created to measure voice selectivity using naturalistic stimuli (*standard sequence*) and to control for low-level acoustic confounds (i.e., frequency content, *scrambled sequences*). If, at the target (voice) rate, the *standard* and the *scrambled* sequences evoke responses that are quantitatively (amplitude of the response) and qualitatively (topographical map, pattern of harmonics) similar, it would indicate that we are interpreting as voice-selective responses that are elicited by frequency content alone. Each stimulation sequence was 64 s long, including 2 s of fade-in and fade-out during which the presentation volume raised gradually from 0 to the maximum at the start of the sequence, and vice versa at the end of the sequence. Fade-in and fade-out were introduced to avoid abrupt movements that the sudden onset of the sequences could have provoked and that would have introduced artefacts in the data. Sequences for both conditions were presented four times in a pseudo-randomized order. Individual sequences with a new pseudo-randomized order of stimulus presentation were generated before testing for each repetition and for each individual participant in an effort to increase generalization; all sequences played during the testing therefore constitute unique exemplars that share presentation parameters (base and target frequencies) and the sounds which were used to build them, but systematically presenting them in different order in each sequence and each participant. During testing, participants were asked to perform an orthogonal non-periodic task: they had to press a button whenever they heard a sound that was presented at a lower volume as compared with the volume of the other sounds in the sequence. Volume reduction was obtained by decreasing the sounds’ root mean square values of a factor of 12.5. Each sequence contained six attentional targets (including both vocal and non-vocal sounds) that were introduced in a pseudo-randomized order (excluding fade-in and fade-out period at the start and the end of the sequence). Participants were required to listen to the sequences and perform the task blindfolded sitting at 90-cm distance from the speakers.

#### EEG acquisition

The EEG was recorded with a Biosemi Active Two system (https://www.biosemi.com/products.htm) with 128 Ag-AgCl active electrodes at a sampling rate of 512 Hz. Recording sites included standard 10–20 system locations as well as intermediate positions (position coordinates can be found at https://www.biosemi.com/headcap.htm). The magnitude of electrode offset, referenced to the common mode sense (CMS), was held below ±50 mV.

#### Analysis

Data analysis was performed using the Letswave5 toolbox (https://github.com/NOCIONS/Letswave5) and the FieldTrip toolbox (Oostenveld et al., 2011) running on MATLAB_R2016b (MathWorks), custom-build scripts in MATLAB_R2016b and RStudio (RStudio Team, 2018).

#### Preprocessing

A fourth order Butterworth bandpass filter with cutoff values of 0.1–100 Hz was applied to the raw continuous EEG data of each participant. Electrical noise at 50, 100, and 150 Hz was attenuated with a FFT multinotch filter with a width of 0.5 Hz. Data were then downsampled to 256 Hz to facilitate data handling and storage. Subsequently, data were segmented into 69 s-long epochs (we will use the term epoch in the analysis sections to refer to the EEG data relative to one sequence of stimulation) to include 2 s before the onset of the fade-in and 3 s after the offset of the fade-out of the stimulation sequences. At this stage, after visual inspection of the data, four subjects were excluded from further analysis as their EEG trace was highly contaminated by artefacts. In the remaining 16 participants, noisy channels were linearly interpolated with the closest neighboring channels (three/four, considering electrodes representing the full area around the interpolated one). This procedure was carried on six participants with no more than 5% of the electrodes (Bottari et al., 2020; Retter et al., 2020) being interpolated in each participant (mean number of channels interpolated considering all 16 participants is 1, range: 0–6; of the channels interpolated across the six participants, six were posterior-occipital electrodes, two central parietal, one central, one temporal, one fronto-temporal, four fronto-central, and one anterior-frontal). The same analyses performed on the 10 participants on which no channel interpolation was performed and on the entire group (with interpolation) led to very similar results. Epochs that presented multiple artefacts after channel interpolation were removed, with no more than one epoch per condition per participant being excluded. All 128 EEG channels were then re-referenced to the common average of all electrodes.

#### Frequency domain analysis

Considering the frequency resolution (1/duration of the sequence) and the frequency of presentation of voices (target frequency), preprocessed data were re-segmented so as to contain an integer of voice presentation cycles to avoid overspill of the target-rate response in the frequency domain. Therefore, epochs were re-segmented excluding fade-in and fade-out and had a final length of 60 s. For each condition and participant separately, epochs were averaged in the time domain to attenuate EEG activity not in phase with the auditory stimulation. A fast Fourier transformation was applied, resulting in amplitude spectra for each channel, condition and subject. Amplitude spectra ranged from 0 to 128 Hz and had a very high resolution of 0.0167 Hz (i.e., 1/60 s), thus allowing to isolate responses at the frequencies of interest and their harmonics. Then, we determined the number of significant harmonics at the group level for target frequency and base frequency: for each condition separately, we computed the grand average across subjects, pooled all channels together and calculated *z* scores on these averaged spectra including as baseline 20 surrounding frequency bins (10 bins on each side excluding the immediately adjacent bins, the local minimum and the local maximum; Retter and Rossion, 2016). Harmonics of the target and base frequencies were considered as significant if their relative *z* scores were higher than 2.32 (i.e., *p* < 0.01, one-tailed, signal > noise). Consecutive significant harmonics were considered, excluding frequencies corresponding to the base-rate responses for the count of target-rate significant harmonics. Responses are represented as topographical head maps summing baseline subtracted amplitude spectra at significant harmonics for target and base frequencies separately, where baseline was calculated as for the calculation of *z* scores. Baseline subtraction before quantification of the response enables to take into account the fact that different frequency bands are differentially affected by noise in EEG recordings (Luck, 2014), with typically higher noise at low frequencies (<1 Hz) and in the α frequency band (8–13 Hz). We also compared the sum of the baseline subtracted amplitude of the significant harmonics as elicited by the *standard* and the *scrambled* sequences (*standard* > *scrambled*, Bonferroni correction for the 128 electrodes). To compute this comparison, we considered the highest number of significant harmonics in any of the two conditions (here, *standard*) knowing that including baseline-subtracted activity at non-significant harmonics to compute the overall response is not detrimental (i.e., adding zeroes). The electrodes that were significant for the *standard* > *scrambled* comparison were considered to define the voice-selective region of interest (ROIvoice).

To assess the robustness of the method to identify voice-selective responses with an even shorter acquisition time, we conducted the same analysis to individuate significant harmonics considering only the responses elicited by the first stimulation sequence for each condition (i.e., 1 min of recording).

Lastly, we performed source localization to identify the generators of the voice-selective response. Source localization was implemented here with Dynamic Imaging of Coherent Sources (DICS; Gross et al., 2001) following the method as described in Popov et al., 2018. Using the cross-spectral density (CSD) calculated at the sensor level, DICS estimates the interaction between sources at a particular frequency: in this case, at the voice presentation frequency (1.333 Hz). This beamformer was chosen since it yields lower localization error despite low SNR when compared with other current density measures (Halder et al., 2019). To attenuate brain activity not in phase with the auditory stimulation, all epochs were averaged in the time domain, then across all subjects for *standard* and *scrambled* sequences separately; consequently, a Fourier transform was applied to compute the CSD. The forward model was computed with the segmentation of the MRI152 template, based on which a headmodel was generated using the boundary element model (BEM), characterizing the current conduction and propagation properties in surfaces of scalp, skull and brain. Sources were placed in the brain part of the volume conduction headmodel with a resolution of 5 mm. Further, we performed a manual co-registration of the headmodel and Biosemi electrode coordinates using rotation, translation and scaling of the electrodes on the scalp to match our best visual estimate. Because of the imprecise co-registration of the forward model, as we did not have individual participants’ MRI and precise electrode location on the scalp, we did not focus on the exact anatomic areas of the brain generators, but limited our attention to compare the voxel-by-voxel activity (i.e., coherence) between the two conditions. A common spatial filter was computed by appending the data of the two conditions to localize each condition. A regularization parameter of 5% was used. Then, we calculated the difference of coherence values between the *standard* and *scrambled* conditions taking the whole brain into consideration to estimate an overall activity at the target frequency. We hypothesized to obtain higher coherence values for the *standard* condition.

Although source analysis is introduced to suggest a link with previous neuroimaging studies, results should be interpreted cautiously, not only because of the indeterminate nature of source localization from scalp voltage potentials but also because we did not collect MRI scans of individual participants, nor the electrode positions on their scalp during the EEG sessions, important elements that would enhance the precision and accuracy of source localization (Akalin Acar and Makeig, 2013). We therefore consider the results of the source localization for visualization purpose only and the main statistical inferences were done on the scalp data.

### Experiment 2 - Voice versus musical instruments controlled for low level acoustic properties

Materials and methods were the same as for experiment 1 unless specified below.

#### Stimuli

Sounds were extracted from a database provided by Agus and collaborators (Goto et al., 2003; Agus et al., 2017). Stimuli were 128 ms long, therefore the base and target frequencies of what we will refer to as *harmonic* sequences were approximately 7.813 Hz and 2.604 Hz, respectively. Vocal stimuli (16 exemplars) consisted in vowels /a/, /e/, /i/ and /o/ sung in the note A3 by two male and two female singers. Non-vocal sounds consisted in 16 different musical instruments playing the note A3 (oboe, clarinet, bassoon, saxophone, trumpet, trombone, horn, guitar, mandolin, ukulele, harpsichord, piano, marimba, violin, viola, and cello, for a total of 16 stimuli). Importantly, vocal and non-vocal sounds implemented for this sequence type were matched for pitch (mean = 223.5 Hz, SD = 7.1 Hz for voices, mean = 220.6 Hz, SD = 1.85 Hz for instruments, Welch’s two-sample *t* test *t*_(17.03)_ = 1.56, *p* = 0.14), spectral center of gravity (Mann–Whitney test, *W* = 107, *p* = 0.45) and HNR (mean = 20.9 dB, SD = 2.5 dB for voices, mean = 18.9 dB, SD = 5.0 dB for instruments, Welch’s two-sample *t* test *t*_(21.8)_ = 1.45, *p* = 0.16; Fig. 1*E*). Pitch, spectral center of gravity and HNR were computed using the Praat software (Boersma, 2001). Stimuli were equalized in overall energy (RMS) and their RMS value was set as to match the energy of the sounds of experiment 1. Sounds were then faded-in and faded-out with 10 ms ramps to facilitate individual sounds segregation and avoid clicking.

#### Procedure

*Harmonic sequences* were generated following the same procedure as reported for experiment 1, presenting sounds one after another with each third sound being a voice. Sequences were 65.5 s long including 2 s of fade-in and fade-out. As in experiment 1, individual sequences were created before testing for each repetition and for each individual participant and included six attentional targets consisting in sounds played at a lower volume. Participants were required to listen to four different *harmonic* sequences and press a button whenever they perceived a sound played in a lower volume sitting blindfolded at 90 cm from the speakers.

#### Analysis

Data were analyzed as in experiment 1 with the epochs being re-segmented from 2.048 s after the onset of the sequences (to exclude fade-in and to start segmenting from the onset of the first sound at 100% volume) and had a final length of 59.9 s to contain an integer of voice presentation cycles and avoid overspill of the target-rate response in the frequency domain. The number of significant harmonics for the responses at the target and base frequencies were calculated as in the previous experiment considering all four stimulation sequences or the first sequence alone. We then performed a region-of-interest analysis by averaging the sum of the baseline subtracted amplitude of the significant harmonics of the target of the electrodes of the ROIvoice as defined in experiment 1 and contrasted the resulting activity against 0.

### Experiment 3 - Behavioral detection of voices

To validate that the sounds presented in the two EEG experiments could effectively be categorized as vocal/non-vocal sounds and to assess the effectiveness of the sound scrambling procedure in altering intelligibility, we conducted a behavioral experiment in which participants had to classify all sounds, presented either in short sequences or in isolation, as vocal or non-vocal sounds.

#### Participants

Sixteen participants (age range 18–26 years, nine female), four of which had previously participated in the EEG experiments, took part in the behavioral experiment. All participants reported normal or corrected to normal vision, normal hearing and no history of psychiatric or neurologic disorders. The experiment was approved by the local ethical committee of the University of Louvain (project 2016-25), all participants provided written informed consent and received financial compensation for their participation.

#### Stimuli

Auditory stimuli were the same as for experiments 1 and 2, all equalized so to have the same overall energy (RMS) and faded-in and -out with 10-ms ramps.

#### Procedure

Participants listened to sounds corresponding to the three sequence types of experiments 1 and 2 (*standard*, *scrambled*, and *harmonic*) that were presented either embedded in short sequences of five sounds (*task sequence*) or in isolation (*task isolation*). For the *sequence* task, short sequences were created so that they could contain either one vocal sound or none (50%/50% of occurrences). In particular, when a vocal sound was presented in a short sequence, it was inserted as the third sound to mimic the structure of sequences implemented for the EEG experiments. For each condition, 80 sequences were created (40 with one voice, 40 without voices), for a total of 240 trials (one short sequence of the behavioral experiment = one trial). In the *isolation* task, for the sequence types *standard* and *scrambled*, we presented 33 vocal and 67 non-vocal sounds extracted randomly for each participant to reproduce the 1:2 ratio of vocal and non-vocal stimuli presented in sequences in the EEG experiment. For the *harmonic* sequence, we presented all 16 vocal and 16 non-vocal stimuli. Sounds from each of the three conditions were presented once in a randomized order, for a total of 132 trials (one sound = one trial). The *sequence* and *isolation* tasks were presented one after another. Each trial consisted in the presentation of one short sequence (sequence task)/one sound (isolation task) after which participants had to indicate whether they heard a voice or not with a button press and a response was required to initiate the following trial. Participants performed the task blindfolded. The experiment was implemented on MATLAB _R2016b (MathWorks) using the Psychophysics Toolbox extensions (Brainard, 1997; Pelli, 1997; Kleiner et al., 2007).

## Results

### Experiment 1 - Voice versus object sounds

We expected clear responses at the base frequency (BF, 4 Hz) and harmonics (multiples of BF: 2BF, 3BF, etc.) for both the *standard* and the *scrambled* sequences, a response at the base frequency reflecting shared processes between all sounds. Then, we predicted to observe, or not, depending on the sequence type, responses at the target frequency (TF, 1.333 Hz) and the harmonics. Critically, a response at the target frequency would arise only if the participant’s brain successfully discriminates human voices from other sounds and generalizes across voices. We predicted that the *standard* sequences would elicit a voice-selective response reflecting selective responses to vocal versus non-vocal sounds. We also hypothesized that if voice-selective responses evoked by the *standard* condition were the mere by-product of frequency content, the *scrambled* condition would have elicited responses quantitatively (in terms of magnitude of the response) and qualitatively (in terms of topographical distribution of the response) similar to the *standard* condition.

#### Base frequency

**Figure 2.**
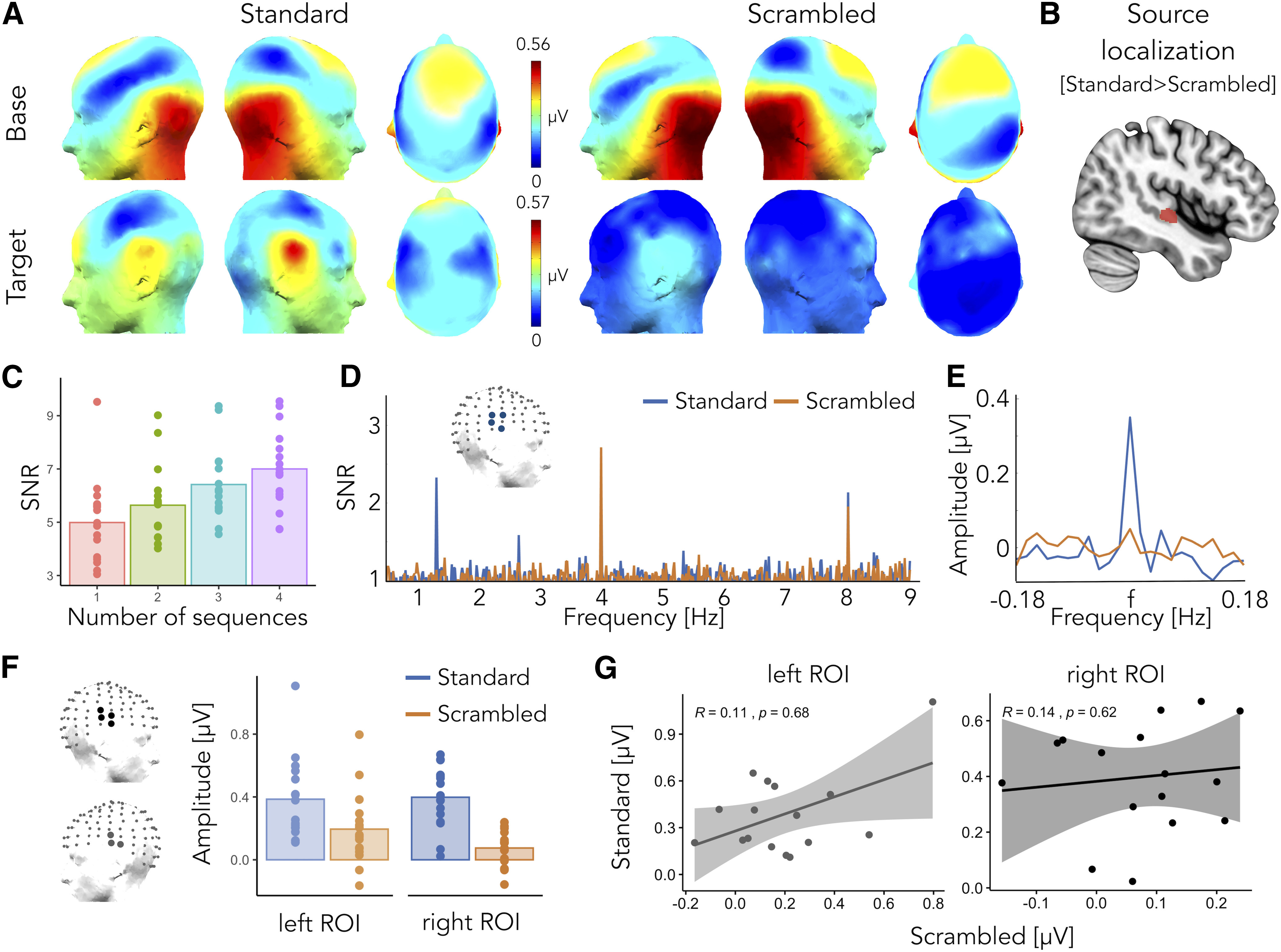
Experiment 1. Voice versus object sounds. ***A***, Responses at the base (top) and at the target frequency (bottom) for the *standard* (left) and *scrambled* (right) conditions as topographic head plots. Topographic head plots were obtained by summing the baseline subtracted amplitude at the significant harmonics. ***B***, Source localization analysis revealed stronger coherence values at the target frequency for the *standard* condition (*standard* > *scrambled*) over right superior and middle temporal areas (voxels with intensity above the 95th percentile are highlighted in red). ***C***, Signal-to-noise ratio (SNR) of voice-selective responses obtained by averaging SNR values of ROIvoice electrodes as a function of number of stimulation sequences/minutes of recording for the *standard* condition. ***D***, SNR averaged spectra of ROIvoice electrodes. ***E***, Sum of the baseline corrected amplitude (considering 10 bins on each side, excluding the immediately adjacent ones) of voice selective responses at the four significant harmonics for the *standard* (blue) and *scrambled* condition (orange). ***F***, Responses at the target frequency for the *standard* (blue) and *scrambled* (orange) conditions as the sum of baseline subtracted amplitude at four harmonics in the left and right ROIs. ***G***, There is no correlation between responses at the left and right ROIs across the *standard* and *scrambled* conditions.

We observed significant responses for the first two harmonics of the base stimulation frequency, i.e., at 4 and 8 Hz, for both the *standard* and *scrambled* sequences. The scalp topographies of the two conditions obtained summing baseline corrected amplitudes at the two significant harmonics did not differ, suggesting that individual sounds were similarly processed between the *standard* and *scrambled* conditions, with responses peaking over central and occipital electrodes (Fig. 2).

#### Target frequency

**Figure 3.**
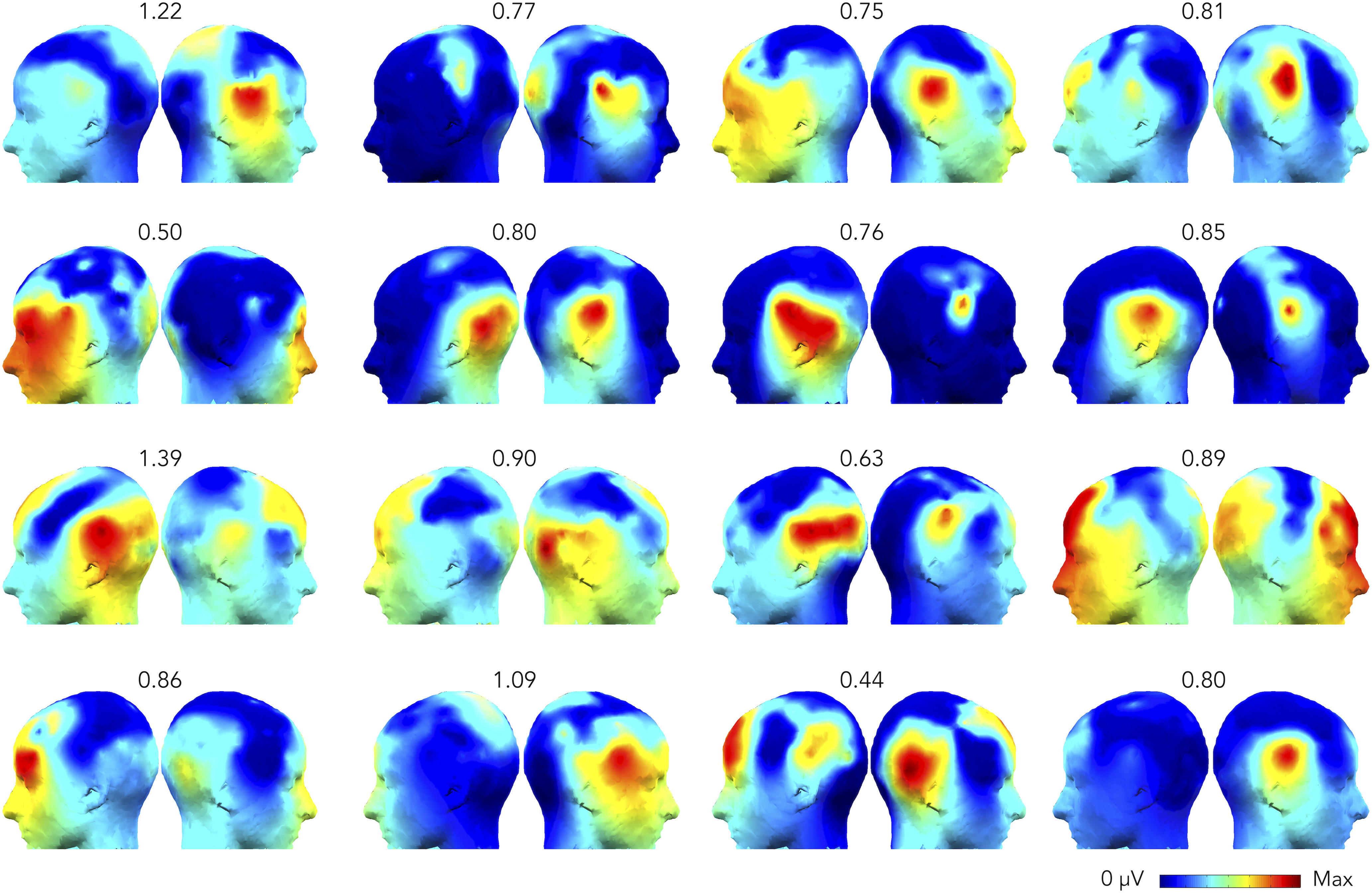
Experiment 1. Responses at the voice presentation frequency for each individual (*standard* condition). Topographic head plots represent the sum of the baseline subtracted amplitude of significant harmonics as identified at the group level. The scale of each head plot ranges from 0 μV to the maximum (reported on top) for each subject.

The *standard* sequence elicited robust voice-selective responses that were significant at the group level for the first four harmonics of the target frequency (at 1.333, 2.666, 5.333, and 6.666 Hz). For the *scrambled* sequence, we observed a weak but significant response only at the first harmonic of the target frequency (at 1.333 Hz). We quantified voice-selective responses as the sum of the baseline subtracted amplitudes of the highest number of significant harmonics in any of the two conditions (here: *standard*, four harmonics) knowing that including baseline-subtracted activity at non-significant harmonics to compute the overall response is not detrimental (i.e., adding zeroes; Retter et al., 2018). For the *standard* condition, voice-selective responses peaked bilaterally at superior temporal electrodes (Fig. 2): TP8h, CP6, T8h, T8 on the right hemisphere and T7h, T7, TP7 on the left hemisphere (contrasting the response against zero, one-sided *t* test, reported p-values are Bonferroni corrected, Cohen’s *d* values are reported referred to as *d*, TP8h: *t*_(15)_ = 6.68, *p* = 0.0005, *d* = 1.67; CP6: *t*_(15)_ = 5.51, *p* = 0.0038, *d* = 1.38; T8h: *t*_(15)_ = 6.89, *p* = 0.0003, *d* = 1.72; T8: *t*_(15)_ = 7.16, *p* = 0.0002, *d* = 1.79; T7h: *t*_(15)_ = 5.62, *p* = 0.0031, *d* = 1.40; T7: *t*_(15)_ = 5.44, *p* = 0.0043, *d* = 1.36; TP7: *t*_(15)_ = 5.56, *p* = 0.0035, *d* = 1.39), the topography of the response being consistent across participants (Fig. 3). No electrode reached significance when we computed the same contrast for the *scrambled* condition. Overall, in regions where the response was peaking for the *standard* condition, the amplitude of the response to voice-scrambled stimuli was of 50.6% of the response to voices for the left ROI (T7h, T7, TP7) and only 18.8% for the right ROI (TP8h, CP6, T8h, T8; Fig. 2*F*). Moreover, the magnitude of the responses to the *standard* and *scrambled* conditions over these two ROIs did not correlate across participants (Spearman’s ρ correlation, left ROI: *r*_s_ = 0.11, *p* = 0.68, right ROI: *r*_s_ = 0.14, *p* = 0.62; Fig. 2*G*). This suggests that, although scrambled voices could elicit a response, frequency content alone was not enough to elicit a voice-selective response as recorded with the *standard* sequence. We further investigated that by comparing the responses elicited at the target frequency by the *standard* and the *scrambled* sequences; although one of the advantages of the FPAS paradigm is that it does not require a direct comparison between conditions, we decided to include this extra step as a proof of principle since we introduce oddball fast periodic stimulation with high-level, naturalistic sounds for the first time here. First, for each condition, participant and electrode separately we summed the responses of the highest number (i.e., four) of significant harmonics in any of the two conditions. Then, comparing the responses for each participant and electrode (*standard* > *scrambled*, paired-sample *t* test, Bonferroni corrected), we isolated four significant superior temporal electrodes over the right hemisphere: TP8h, CP6, C6 and T8 (TP8h: *t*_(15)_ = 6.21, *p* = 0.0011, *d* = 1.55; CP6: *t*_(15)_ = 5.02, *p* = 0.0097, *d* = 1.26; C6: *t*_(15)_ = 4.71, *p* = 0.0179, *d* = 1.18; T8: *t*_(15)_ = 4.60, *p* = 0.0223, *d* = 1.15, Bonferroni corrected). Non-parametric testing (Winkler et al., 2014) of the *standard* > *scrambled* comparison led to very similar results and the choice of a one-sided comparison was made with the a priori assumption that the *scrambled* condition would have elicited a target response as high- if voice responses could have been explained by frequency content alone- or smaller than the one elicited by the *standard* sequence. Finally, a region-of-interest was defined considering the four electrodes identified as above (ROIvoice; Fig. 2).

To assess whether FPAS was a suitable tool to investigate voice-selectivity at the individual level, we calculated whether these responses were significant in every subject (Liu-Shuang et al., 2016). For each participant, we considered the amplitude spectrum and we pooled together all channels. We chunked epochs that were centered around the harmonics of the target frequency that were significant at the group level (1.333, 2.666, 5.333, and 6.666 Hz) and that contained 11 frequency bins on each side. We then summed these epochs together and computed the *z* scores at the target frequency considering as baseline the 20 surrounding frequency bins (10 on each side, excluding the immediately adjacent bin). Considering as significant responses whose relative *z* scores was higher than 1.64 (i.e., *p* < 0.05, one-tailed, signal > noise), we were able to find significant voice-selective responses in 13 out of 16 participants (with only 4 min of recordings).

To assess the robustness of the method with an even shorter acquisition time, we then performed the same analytical steps done to calculate the number of significant harmonics at the group level considering the first sequence of recording only. For the *standard* sequence, we were able to identify voice-selective responses that were significant at the group level even with 1 min of recording only (one significant harmonic: 1.333 Hz). The signal-to-noise ratio (SNR) of the response at right superior temporal electrodes (ROIvoice) as a function of number of stimulation sequences (i.e., minutes of recordings, as each sequence is 1 min long) is presented for visualization purposes in Figure 2*C*.

A stronger voice-selective response in the *standard* condition was observed when compared with the *scrambled* condition at the source level as well. After implementing source localization, the final voxel coherence values were compared for the two conditions (*standard* > *scrambled*) and interpolated to the MRI152 template. Then, the voxels with intensity above the 95th percentile were selected, as illustrated in Figure 2*B*. The maximum value obtained of 0.32 (min-max range: [0, 1]) indicates voxel preference for voice presentation frequency (i.e., 1.333 Hz) for the *standard* over the *scrambled* sequence. Despite the imprecise source localization because of the limitations outlined in the method section (lack of individual coregistration between electrodes position and brain anatomy), the position of the source reconstructed effect of interest (Fig. 2*B*) lies in the vicinity of the known location of TVAs (Belin et al., 2000, 2002) and are in line with results showing that right anterior STS regions respond more strongly to non-speech vocal sounds than their scrambled versions (Belin et al., 2002).

### Experiment 2 - Voice versus musical instruments controlled for low level acoustic properties

We expected to observe a response at the base presentation frequency and its harmonics, this response reflecting processes that are shared among vocal and non-vocal sounds. More crucially, we predicted that, if voice-selectivity is not solely driven by harmonicity and/or pitch of the sounds, we would observe a target-rate response to the *harmonic* sequences, as in these sequences, vocal and non-vocal sounds did not differ for these low-level acoustic features.

#### Base frequency

**Figure 4.**
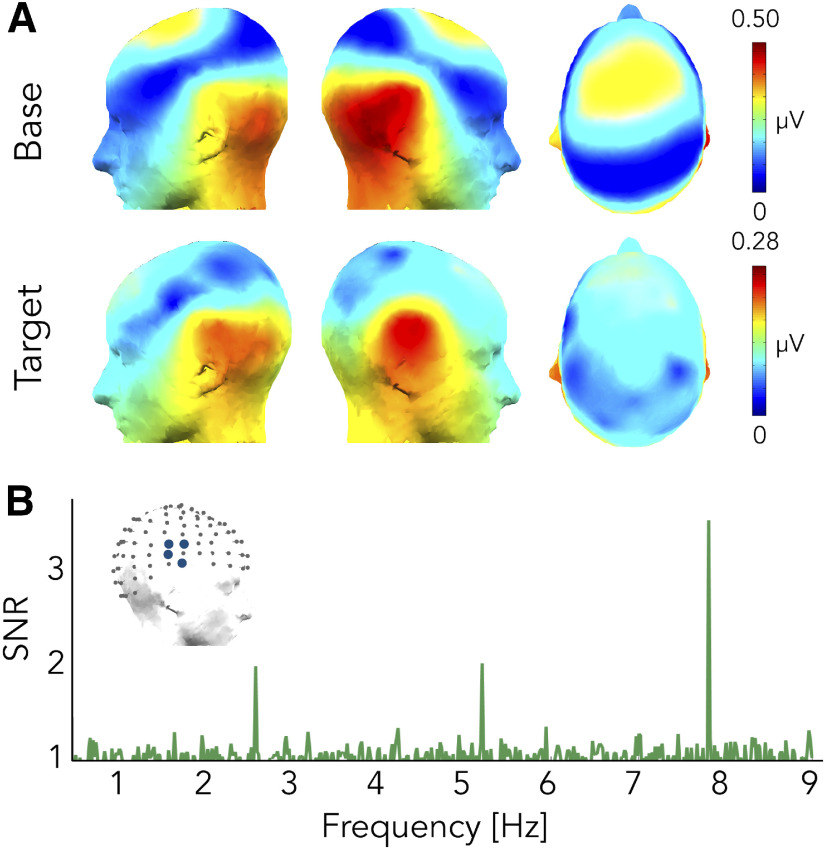
Experiment 2. Voice versus musical instruments. ***A***, Responses at the base and at the target frequency as topographic head plots. Topographic head plots were obtained by summing the baseline subtracted amplitude at the significant harmonics. ***B***, SNR averaged spectra of ROIvoice electrodes.

There was a significant base-rate response for the first two harmonics: at 7.813 and 15.625 Hz, with responses peaking over central and occipital electrodes (Fig. 4). The topography of the response was qualitatively similar to the base-rate responses obtained in experiment 1.

#### Target frequency

We observed a significant voice-selective response for the first two harmonics: at 2.604 and 5.208 Hz. Voice-selective responses peaked over central and superior temporal electrodes bilaterally, the scalp distribution of the response being highly similar to the voice-selective response found in the *standard* sequence (Fig. 4) and being reliable across participants. We then performed a ROI analysis considering the right superior temporal electrodes (ROIvoice) as defined in experiment 1. Baseline subtracted amplitudes at the two significant harmonics were summed for each electrode individually and the resulting responses at the electrodes of the ROIvoice were then averaged together for every participant. One data point was excluded as it deviated more than three standard deviations from the mean of the group and responses were significant against zero (mean = 0.152 μV, SD = 0.087 μV, *t*_(14)_ = 6.78, *p* = 4.436 × 10^−6^, *d* = 1.75, one-tailed *t* test).

Voice-selective responses were already present after 1 min of recording, showing the resilience of FPAS even with very short acquisition time (one harmonic significant, the second: 5.208 Hz, with 2 min of recording the first two harmonics reached significance at the group level; Fig. 4).

As for the *standard* condition, we then assessed significance of voice-selective responses at the individual level with the same procedure. A total of 15 out of 16 participants showed a significant response (*z* score higher than 1.64, i.e., *p* < 0.05, one-tailed, signal > noise).

### Experiment 3 - Behavioral detection of voices

**Figure 5.**
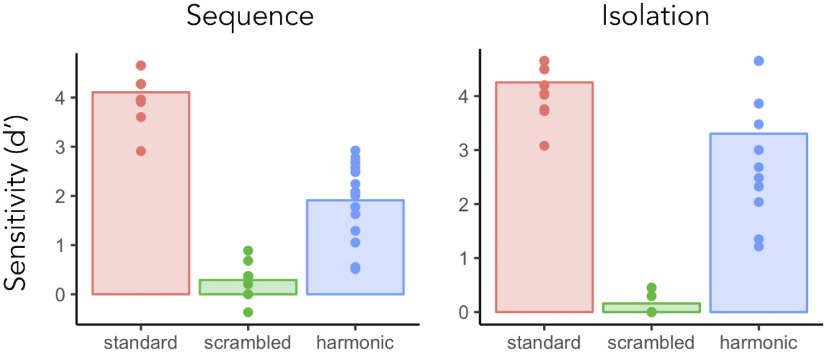
Experiment 3. Behavioral detection of voices. Sensitivity indices for the *sequence* (left) and *isolation* (right) tasks, for the three conditions. The bar plots represent the group average of d′, each dot represents an individual participant’s d′ score.

Responses to the *sequence* and *isolation* tasks were analyzed according to signal detection theory. Specifically, sensitivity indices related to the participants’ ability to detect a voice when present was assessed using d-prime (Fig. 5); d-prime (d′) constitutes an unbiased quantification of performance in detection tasks as it takes into account both hits and false alarms (Tanner and Swets, 1954; Macmillan and Creelman, 2004). Data points were considered as outliers when deviating from the mean of the group of more (or less) than three times the standard deviation of the group in at least one condition/task, leading to the exclusion of one participant. We first checked whether performance was above chance (d′ = 0) for each condition and for the *sequence* and the *isolation* task separately. Participants performed above chance for all conditions in the *sequence* (*t* test, one-tail, *standard*: d′ values mean = 4.11, SD = 0.45, *t*_(14)_ = 35.30, *p* = 2.2 × 10^−15^, *d* = 9.11; *scrambled*: mean = 0.29, SD = 0.30, *t*_(14)_ = 3.75, *p* = 0.001, *d* = 0.97; *harmonic*: mean = 1.91, SD = 0.77, *t*_(14)_ = 9.58, *p* = 7.8 × 10^−8^, *d* = 2.47) and in the *isolation* task (*t* test, one-tail, *standard*: mean = 4.25, SD = 0.46, *t*_(14)_ = 35.71, *p* = 1.8 × 10^−15^, *d* = 9.21; *scrambled*: mean = 0.16, SD = 0.21, *t*_(14)_ = 2.97, *p* = 0.005, *d* = 0.77; *harmonic*: mean = 3.30, SD = 1.24, *t*_(14)_ = 10.29, *p* = 3.3 × 10^−8^, *d* = 2.66). Statistical comparisons of conditions were then performed separately for the two tasks using repeated measures ANOVAs. Whenever Mauchly’s test indicated that the assumption of sphericity had been violated, we applied a Greenhouse–Geisser correction to the degrees of freedom. For the *sequence* task, we found a significant effect of condition (*F*_(2,28)_ = 207.39, *p* = 1.6 × 10^−17^, η^2^ = 0.898) and *post hoc* pairwise comparisons showed that all conditions differed one from another (*standard* vs *scrambled*, *p* < 2 × 10^−16^; *standard* vs *harmonic*, *p* = 7.1 × 10^−9^; *scrambled* vs *harmonic*, *p* = 1.5 × 10^−6^; Bonferroni corrected). The same pattern of performance was found when sounds were presented in isolation (*F*_(1.20,16.86)_ = 134.23, *p* = 6.8 × 10^−10^, η^2^ = 0.845; pairwise comparisons: *standard* vs *scrambled*, *p* < 2 × 10^−16^; *standard* vs *harmonic*, *p* = 0.038; *scrambled* vs *harmonic*, *p* = 2.7 × 10^−7^; Bonferroni corrected). Although performance was above chance for the scrambled sounds, d′ scores were significantly lower than the one achieved in the *standard* condition (Fig. 5), suggesting that our frequency scrambling effectively disrupted the intelligibility of the sounds.

## Discussion

Similarly to the issue of category-selectivity in human visual cortex (Rice et al., 2014; Bracci et al., 2017; Peelen and Downing, 2017), studies have attempted to determine whether category-selectivity in auditory cortices is mostly driven by a biased tuning toward low-level acoustic features or whether categorization responses go beyond the acoustic properties of sound and therefore represent a more abstract representation of sound category (Leaver and Rauschecker, 2010; Giordano et al., 2013). In fact, as sounds tend to be acoustically similar within an auditory category and dissimilar between categories, differences in cortical responses could be a mere reflection of different acoustic properties across categories of sounds (Staeren et al., 2009; Ogg et al., 2020). One approach to address this question is to test for low-level featural coding. However, although studies using artificial stimuli allow for a careful control of low-level acoustic properties (Patterson et al., 2002; Warren et al., 2005; Lewis et al., 2009), they usually lack ecological validity and underestimate the issue of stimulus-driven response correlation (Norman-Haignere and McDermott, 2018). Moreover, artificial stimuli may fail in eliciting brain activation in a (behaviorally) relevant way, as evidenced by a study revealing different tonotopic maps obtained with natural sounds and pure tones (Moerel et al., 2012).

In this study, we developed a FPAS paradigm as a powerful mean to investigate voice-selectivity in the brain with the aim of disentangling between a categorization response to voices and the contribution of low-level acoustic features that are typical of voices (e.g., high harmonicity, characteristic frequency ranges, specific change of energy over time) with EEG. The frequency constraint of this approach allowed us to individuate robust voice-selective responses objectively, at known stimulation frequencies, and automatically: not only participants did not have to overtly respond to voices, thus avoiding the contamination of the response from attentional and decisional processes (Levy et al., 2003; Von Kriegstein et al., 2003), but also no subtraction between responses elicited by different auditory categories was required. That is, while traditional M/EEG approaches investigate whether there are differences in isolated responses elicited by different classes of stimuli (Levy et al., 2003; Murray et al., 2006; Charest et al., 2009; De Lucia et al., 2010), in our paradigm, this differentiation is implicit. Moreover, the relatively low-speed presentation of stimuli required by many traditional experimental paradigms precludes from the possibility of presenting a high number of stimuli that could be representative enough of an auditory category and therefore responses observed to such a subset of voice samples might not reflect a generalized response to voices (Giordano et al., 2013). In addition, low-speed presentation of stimuli might fail in fully characterizing voice processes as they occur in daily-life, since we are experts in extracting voice features almost effortlessly in highly dynamic acoustically changing environments.

For a voice-specific response to be captured with the FPAS paradigm, two processes need to occur: the brain has to concurrently discriminate voices from non-vocal sounds and to generalize this selective response across diverse vocal samples for a response at the target (i.e., voice presentation) rate to occur. FPAS therefore not only allows to characterize a general voice-selective response, as it is elicited by heterogeneous vocal samples and not from a specific subset of those, but also not to cancel out processes elicited by voices that are shared by other sound categories. This can be accomplished in a very short acquisition time (i.e., 4 min or even less) and with a high SNR (up to 7, or 600% of amplitude increase with four stimulation sequences; Fig. 2*C*).

To isolate voice-selective responses that could not be merely explained by low-level acoustic features, we implemented two EEG experiments using the FPAS principle. In the first experiment, participants were presented with two different types of FPAS sequences. In the *standard* sequence, vocal and non-vocal samples were selected to be as heterogeneous as possible to represent the high variability of sounds of a given category (e.g., voices from speakers from different ages, sex, emotional state with speech and non-speech vocalizations) as encountered in natural environment, as well as to minimize the potential contributions of low-level acoustic features in the voice-selective response (controlling by variability). Specifically, low level acoustic properties would have to vary periodically to participate in the target-specific responses, and the high variability of the voice samples adopted should prevent that. These sounds were then scrambled using small frequency bins (after FFT) to generate a sequence in which scrambled sounds had similar frequency content as the original stimuli (Fig. 1*C*) but were not recognizable anymore (Fig. 5). We measured robust voice-selective responses at superior temporal electrodes that were significant in the vast majority of the participants with only 4 min of recording (Fig. 2). Moreover, these responses remained significant at the group level when restricting the analysis to the first minute of recording only, highlighting the robustness of the method even with an extremely short acquisition time. Voice-selective responses could not have been explained by frequency content alone: although one of the advantages of FPAS is that it does not require an explicit comparison across conditions, here, as an extra proof of principle, we compared the responses elicited by the *standard* and *scrambled* sequences, highlighting a response expressing over right superior temporal electrodes. This preference also emerged at the source space: although source localization as implemented in our study presents limitations that could hinder the accuracy of our results (see Materials and Methods), we localized a *standard* > *scrambled* preference over the right superior temporal gyrus and sulci. The observed regions lie in the proximity of the TVAs and are in line with results showing that the right anterior STS regions respond more strongly to non-speech vocal sounds than their scrambled versions (Belin et al., 2002).

It has to be noted that the *scrambled* condition elicited a weak but yet significant response at the (scrambled) voice presentation rates: we think there might be two, not mutually exclusive, explanations for this weak response. First, this response might be because of the low-level acoustic features shared by voices and scrambled voices (i.e., frequency content): preferential response biases toward acoustic features peculiar of voices have been observed in voice-selective regions (Moerel et al., 2012) and, despite the fact that voice-selective responses elicited by the *standard* condition could not be well explained by low-level acoustic features alone, these features may nevertheless weakly contribute to the response. Second, although the scrambling procedure disrupted the intelligibility of the sounds, with original sounds being more accurately recognized as voices or not than scrambled sounds, participants’ performance in categorizing scrambled sounds was above chance level, suggesting that some residual sound recognizability might have still been present.

To further investigate the nature of voice-selective responses, we designed a second FPAS experiment using sequences built from sung vowels and musical instruments that were matched in terms of pitch, spectral center of gravity and HNR. The observation of robust voice-selective responses despite controlling for the above-mentioned acoustic features speaks in favor of a categorization response to voices that is at least partially independent from some of the most intrinsic acoustic features of voices. The similar scalp topographies elicited by the *standard* and *harmonic* sequences suggest a similarity between the selective responses to voices elicited in the two conditions. However, the voice-selective response in the *standard* sequences was larger in amplitude as compared with the response of the *harmonic* sequence. Different factors might account for this difference. First, the higher amplitude of response observed in the *standard* sequence could be due to voices being more easily discriminated from non-vocal sounds, as pointed out by the behavioral data. In fact, even if an overt response to voices is not necessary to elicit a target rate specific response, the easiness at which a voice is perceived could have impacted the amplitude of brain responses. Second, while vocal stimuli of the *harmonic* sequence were accurately chosen to be matched in acoustic properties with the sounds of musical instruments, it could be argued that those vocal stimuli represent a subset of voices, and thus that the strongest response in the *standard* condition reflects the response to a more heterogeneous and representative sample of voices of that condition. The choice of the frequencies of stimulation could also have impacted on the magnitude of the responses (Regan, 1966; Ding et al., 2006; Retter et al., 2020), while all parameters proved effective in eliciting target-rate responses, further studies would be needed to indicate which parameters are optimal to capture voice-selective responses, as addressed in the case of the implementation of the frequency tagging approach in high-level vision (Alonso-Prieto et al., 2013; Retter et al., 2020). Finally, systematic differences in low-level acoustic features between vocal and non-vocal sounds potentially present in the *standard* sequence could have boosted the categorization response to voices. In fact, feature dependence is not in conflict with a categorical coding hypothesis (Bracci et al., 2017), and preferential response biases observed in voice regions to specific acoustic features such as low frequencies (Moerel et al., 2012), harmonic structure (Lewis et al., 2009), and spectrotemporal modulations (Santoro et al., 2017), could serve as a scaffold or facilitate a higher level categorical responses to voices.

In summary, we objectively defined human voice-selective responses independent from low-level acoustic cues that are characteristic of voices with high SNR and in a very short acquisition time using an original FPAS approach. Although it is possible that other acoustic features that were not explicitly controlled for might be at the origin of the recorded responses, the nature of the FPAS design makes it unlikely, as said features should be systematically present in all voices and absent in non-vocal sounds. In other words, activity in voice‐selective regions could not be only, or even substantially, accounted for by any basic acoustic parameter tested.

While the goal of the current study was to investigate the existence of a voice-selective response partially independent from some acoustic features, the FPAS paradigm we developed could be a valuable tool for the study of auditory perceptual categorization in the human brain, extending to other categories. Moreover, the high SNR of this technique achievable in a very short acquisition time (i.e., significant responses were observed with 1 min of recording) and the fact that no overt response to specific stimuli is required, makes this approach highly promising for studying voice perception in children and clinical populations. Our findings therefore advance our understanding of voice-selectivity in the brain and our method provides an essential foundation for understanding its development in typical and atypical populations.

## Synthesis

Reviewing Editor: Morgan Barense, University of Toronto Decisions are customarily a result of the Reviewing Editor and the peer reviewers coming together and discussing their recommendations until a consensus is reached. When revisions are invited, a fact-based synthesis statement explaining their decision and outlining what is needed to prepare a revision will be listed below. The following reviewer(s) agreed to reveal their identity: Philippe Albouy, Davide Bottari.

REVIEWER 1:

In this study, the authors used a frequency tagging procedure to study if voice selective areas could be identified with EEG data.

In experiment 1 they used heterogeneous vocal sounds (to minimize the contributions of low-level acoustic features in the voice-selective response ) embedded in sequences of heterogeneous non-vocal samples (frequency of sound presentation 4Hz - target frequency for vocal sound 1.33Hz -) to show that electrodes over the temporal regions are entrained at the target frequency. This effect was not present for scrambled sounds.

Then, to confirm that voice-selective response can be elicited for voices when compared to another sound category matched for some low-level acoustical features, they performed a second experiment using sequences built from sung vowels and musical instruments that were matched in terms of pitch, spectral center of gravity and harmonicity to noise ratio.

They observed similar robust voice-selective responses in this experiment, and the authors interpret this effect as being in favor of a categorization response to voices that is at least partially independent from some of the most intrinsic acoustic features of voices.

This is an interesting study, well written and well performed, I have few comments before recommending it for publication.

- Title: The term “voice-selective electrophysiological response” is confusing, the frequency of the response is defined by the frequency of stimulation. The authors did not identify ongoing (self-generated) electrophysiological markers related to voice processing. Please consider revising the title.

- As 128 channels have been recorded, I strongly recommend the authors to perform source reconstruction of the EEG data. This will allow identifying the generators of the response and will allow making stronger links with previous neuroimaging studies.

- I don't consider the use of frequency tagging as a new methodological advance, as this procedure has been used by plenty of research groups, in multiple domains and is well established as powerful procedure. I think the main contribution of this study concerns the investigation of whether the observed selectivity can be explained by specific acoustic properties of voices or represent higher-level categorization response derived from experience. In think the abstract and introduction should be modified to insist on the fact that this study suggests that this selective response is partially independent of low-level auditory properties.

- Could you justify why the sounds have been presented with speakers? This does not seem conventional

- Less than 5% of the electrodes have been interpolated in each participant. This method is a bit controversial as it estimates data based on surrounding electrodes. Could the authors report the results after removing the bad channels? Details about the position of the electrodes removed should be also added.

REVIEWER 2:

The manuscript “A robust voice-selective electrophysiological response in the human brain as revealed by fast periodic auditory stimulation” submitted to eNeuro aimed at characterizing brain response specific for voice perception and to disentangle high-order voice categorization process from low acoustic features dependencies. To this aim, authors implemented a new methodology of investigation, at least in the context of voice perception, based on EEG frequency tagging. This allowed to record objective and automatic selective responses generalized to voice sounds, thus disregarding identity, gender, age, emotion, and accent of the speaker. In order to assess the role of low-level features, they implemented a scrambling procedure to create scrambled sounds matched by frequency and spectral content to voice ones. Moreover, to further understand the dependencies of the process from low level features, they also implemented a second experiment in which target sound was matched to standard ones by several main properties (e.g., pitch, HNR).

The aim of the study is well defined. I particularly appreciate the novelty of the methodology in the context of the experimental question and the translation of FPVA into FPAS.

However I have some concerns which I am confident the authors will address in a revised version of the manuscript

1) The authors dedicated consistent effort to statistically assess which harmonics are significant against the noise. The same approach has been consistently used in other papers on FPVS. For each condition, the authors computed the grand average across subjects, and then across all pooled channels calculated z-scores on averaged spectra using as noise 20 surrounding frequency bins. As arbitrary statistical threshold, the authors used z-scores higher than 2.32 (i.e. p<0.01, 1-tailed, signal > noise).

However, it is opinion of the present reviewer, that the same methodological attention was not ensured to identify which electrodes were statistically significant for the comparison standard - scrambled by contrasting it against zero (despite the Bonferroni correction for the N of electrodes). As this study mostly aims to represent a methodological advancement with regard to the extraction of voice selective responses, I think it would be more appropriate to (1) directly compare standard vs. scrambled in a data driven manner and (2) most importantly, perform the subtraction standard - scrambled, and test that response against a null distribution (e.g. permutation test; e.g. Winkler et al., 2014 Neuroimage), not against zero (the SD of the two distributions, if one is made of zeros, are critically different; moreover if values of standard - scrambled are all positive there is no point in testing it statistically against zero).

2) In the manuscript it is often referred to the objectivity of the measure as it does not imply subtraction across conditions. However, the crucial comparison is standard - scrambled. This logical inconsistency leaves this reader rather puzzled.

3) It is reported that following the scrambling procedure stimuli are not recognizable any more. Is there any behavioural data in support this claim? This would be of help to interpret what participants are perceiving.

4) More on the previous point, the first harmonic is significant even for the scrambled condition. This might reveal that participants were to some extents discriminating between base and target frequency even for the scrambled condition. The authors should provide deeper comments on this, especially if no measure of recognizability (see point 3) is available.

5) At least for the group level, point of views of topo plots should be the same for base and target frequency (Figure 2 and 3) to appreciate how the topography changes.

6) Minor: the mixed use of epoch, trial and sequences is confounding.

## Author Response

Modifications made to manuscript number eN-NWR-0471-20 now titled “A robust voice-selective response in the human brain as revealed by electrophysiological recordings and fast periodic auditory stimulation”.

We wish to thank the editor and the reviewers for taking their time to read our manuscript thoroughly and for providing insightful comments that helped us to significantly improve the quality of the manuscript. In the following section, the comments are presented in bold followed by our responses.

Point-by-point reply:

REVIEWER 1:

In this study, the authors used a frequency tagging procedure to study if voice selective areas could be identified with EEG data.

In experiment 1 they used heterogeneous vocal sounds (to minimize the contributions of low-level acoustic features in the voice-selective response ) embedded in sequences of heterogeneous non-vocal samples (frequency of sound presentation 4Hz - target frequency for vocal sound 1.33Hz -) to show that electrodes over the temporal regions are entrained at the target frequency. This effect was not present for scrambled sounds.

Then, to confirm that voice-selective response can be elicited for voices when compared to another sound category matched for some low-level acoustical features, they performed a second experiment using sequences built from sung vowels and musical instruments that were matched in terms of pitch, spectral center of gravity and harmonicity to noise ratio.

They observed similar robust voice-selective responses in this experiment, and the authors interpret this effect as being in favor of a categorization response to voices that is at least partially independent from some of the most intrinsic acoustic features of voices.

This is an interesting study, well written and well performed, I have few comments before recommending it for publication.

We thank the reviewer for the positive evaluation of our work.

- Title: The term “voice-selective electrophysiological response” is confusing, the frequency of the response is defined by the frequency of stimulation. The authors did not identify ongoing (self-generated) electrophysiological markers related to voice processing. Please consider revising the title.

As the reviewer pointed out, we are indeed not identifying an ongoing self-generated electrophysiological response to voices: the brain responses we recorded with our paradigm are triggered by an external periodic stimulation, whose parameter define the frequency of the response. According to the comment of the reviewer, we have changed the title our manuscript to: “A robust voice-selective response in the human brain as revealed by electrophysiological recordings and fast periodic auditory stimulation”.

- As 128 channels have been recorded, I strongly recommend the authors to perform source reconstruction of the EEG data. This will allow identifying the generators of the response and will allow making stronger links with previous neuroimaging studies.

In accordance with the recommendation of the reviewer, we have now performed source reconstruction of the EEG data. Importantly, since source localization was not an initial objective of our study, we did not have the MRI of our participants and did not have a 3D head-model of the position of electrodes in relation to the head-shape of each participant. This information is important for accurate individual co-registration to enhance the precision and accuracy of source reconstruction from EEG data (Akalin Acar and Makeig, 2013).

Instead, the forward model was computed with the segmentation of the MRI152 template, based on which a headmodel was generated using the boundary element model (BEM), characterizing the current conduction and propagation properties in surfaces of scalp, skull and brain. Sources were placed in the brain part of the volume conduction headmodel with a resolution of 5 mm. Further, we performed a manual co-registration of the headmodel and Biosemi electrode coordinates using rotation, translation and scaling of the electrodes on the scalp to match our best visual estimate.

Source localization was implemented with Dynamic Imaging of Coherent Sources (DICS, Gross et al., 2001) following the method as described in Popov et al., 2018. Using the cross-spectral density (CSD) calculated at the sensor level, DICS estimates the interaction between sources at a particular frequency: in this case, at the voice presentation frequency (1.33 Hz). This beamformer was chosen since it yields lower localization error despite low SNR when compared to other current density measures (Halder et al., 2019). To attenuate brain activity not in phase with the auditory stimulation, all epochs were averaged in the time domain, then across all subjects for standard and scrambled sequences separately; consequently, a Fourier transform was applied to compute the CSD. A common spatial filter was computed by appending the data of the 2 conditions, to localize each condition (standard and scrambled sequences). Regularization parameter of 5% was used. After implementing source localization, the final voxel coherence values were compared for the two conditions (i.e. standard > scrambled), taking the whole brain into consideration, to estimate an overall activity at the target frequency. We hypothesized to obtain higher coherence values for the standard condition. The result was interpolated to the MRI152 template and the voxels with intensity above the 95th percentile were selected for visualization purpose (figure below and now figure 2B in the manuscript). The source localization indicates voxel preference at the voice presentation frequency (i.e. 1.33 Hz) for the standard over the scrambled sequence.

Despite the somehow imprecise source localization due to the limitations of the forward model, the position of the source reconstructed effect of interest lies in close vicinity of what we know to be the location of TVAs (Belin et al, 2001) and are in line with results showing that right STS regions responded more strongly to non-speech vocal sounds than their scrambled versions (Belin et al., 2002).

Following the recommendation of the reviewer, the result of this source localization analysis is now reported in Figure 2B (see also in Materials and methods, paragraph 2.1.7; in Results, paragraph 3.1.2; in Discussion, paragraph 4).

- I don’t consider the use of frequency tagging as a new methodological advance, as this procedure has been used by plenty of research groups, in multiple domains and is well established as powerful procedure. I think the main contribution of this study concerns the investigation of whether the observed selectivity can be explained by specific acoustic properties of voices or represent higher-level categorization response derived from experience. In think the abstract and introduction should be modified to insist on the fact that this study suggests that this selective response is partially independent of low-level auditory properties.

Thank you for this comment. We agree that the originality of this study does not only lie in the use of frequency tagging itself since this procedure, as rightly pointed out by the reviewer, has been used by many research groups and to study diverse cognitive processes. However, our contribution partially resides in the fact that we are using frequency tagging to investigate high-level auditory processes (voice categorization) with complex and naturalistic sounds for the first time (at least to our knowledge). In fact, if frequency tagging has been used in many studies in vision to study low level as well as higher level functions (such as face and word processing, just to name a few); this technique has been mainly used to study “low-level” information processing (e.g. pitch) in audition. Here, we are using this powerful procedure to study higher-level auditory processes with naturalistic complex sounds for the first time. This more “methodological” contribution is also highlighted by the reviewer #2: “The aim of the study is well defined. I particularly appreciate the novelty of the methodology in the context of the experimental question and the translation of FPVA into FPAS”. For these reasons, we hope that the reviewer will agree that our manuscript still promotes such more “methodological” contribution, in addition to its conceptual advance.

That being said, and in accordance with the reviewer’s comment, we agree that the main contribution of our study remains “conceptual”: the observed selectivity to voices represent higher-level categorization response that is at least partially independent from the specific acoustic properties of voices. We therefore modified the abstract accordingly. In the introduction we explain more clearly how the FPAS technique is at the service of our conceptual goal (tag high-level voice categorization independently of acoustic features). Moreover, the conceptual advances of our research are more broadly elaborated in the discussion section.

- Could you justify why the sounds have been presented with speakers? This does not seem conventional

The choice to use speakers was driven by our effort to create a stimulation that could be more ecological and that could be as close as possible to social interaction in daily life. In fact, we usually interact with people located in the external space, where we can rely on extra cues such as spatial location.

We have recently piloted a similar experimental paradigm that included auditory stimulation as created in our experiment 1 in the manuscript (standard sequence) and we had the opportunity to record data from two participants using two different setups: with speakers (as in the manuscript) and with earphones, and we observed almost identical results (see figure below).

Voice selective responses as elicited by FPAS with a paradigm similar to the one reported in the manuscript (same parameters as standard sequence) delivering sound through speakers (left) and earphones (right). Topographic head plots were obtained by averaging baseline subtracted amplitudes on two participants and summing the amplitude at four harmonics.

- Less than 5% of the electrodes have been interpolated in each participant. This method is a bit controversial as it estimates data based on surrounding electrodes. Could the authors report the results after removing the bad channels? Details about the position of the electrodes removed should be also added.

We have gone back to this section in the manuscript and we think that the wording, that has now been changed in the text (see paragraph 2.1.6), might have been confusing. In fact, channel interpolation has not been performed in every participant but only in the participants presenting noisy channels and, when interpolation was performed, it was carried on a maximum of 5% of the channels (that is on a maximum of 6/128 channels). This procedure was only carried on six of the sixteen participants. This procedure is actually pretty standard and was for instance used in recent papers using FPVS (see Bottari et al., 2020, Retter et al., 2020 for an example). More details have now been added in the manuscript.

Unfortunately, it is not possible to show group results after removing bad channels because the channels interpolated (if any) vary across the subjects and therefore we cannot average across participants unless they all have the same channels in the data. Nevertheless, in order to report data with no channel interpolation, we performed the same analysis carried out in our manuscript on the 10 subjects on which no channel interpolation was done and, as one can appreciate in the figure below, the results are almost identical to the results from the complete group sample with interpolation (figures below).

A sentence has now been added in the manuscript saying that performing analyses on the 10 subjects without interpolation leads to results almost similar to those obtained with the entire group including interpolation.

-

REVIEWER 2:

The manuscript “A robust voice-selective electrophysiological response in the human brain as revealed by fast periodic auditory stimulation” submitted to eNeuro aimed at characterizing brain response specific for voice perception and to disentangle high-order voice categorization process from low acoustic features dependencies. To this aim, authors implemented a new methodology of investigation, at least in the context of voice perception, based on EEG frequency tagging. This allowed to record objective and automatic selective responses generalized to voice sounds, thus disregarding identity, gender, age, emotion, and accent of the speaker. In order to assess the role of low-level features, they implemented a scrambling procedure to create scrambled sounds matched by frequency and spectral content to voice ones. Moreover, to further understand the dependencies of the process from low level features, they also implemented a second experiment in which target sound was matched to standard ones by several main properties (e.g., pitch, HNR).

The aim of the study is well defined. I particularly appreciate the novelty of the methodology in the context of the experimental question and the translation of FPVA into FPAS.

Thank you for the positive evaluation.

However I have some concerns which I am confident the authors will address in a revised version of the manuscript

1) The authors dedicated consistent effort to statistically assess which harmonics are significant against the noise. The same approach has been consistently used in other papers on FPVS. For each condition, the authors computed the grand average across subjects, and then across all pooled channels calculated z-scores on averaged spectra using as noise 20 surrounding frequency bins. As arbitrary statistical threshold, the authors used z-scores higher than 2.32 (i.e. p<0.01, 1-tailed, signal > noise).

However, it is opinion of the present reviewer, that the same methodological attention was not ensured to identify which electrodes were statistically significant for the comparison standard - scrambled by contrasting it against zero (despite the Bonferroni correction for the N of electrodes). As this study mostly aims to represent a methodological advancement with regard to the extraction of voice selective responses, I think it would be more appropriate to (1) directly compare standard vs. scrambled in a data driven manner and (2) most importantly, perform the subtraction standard - scrambled, and test that response against a null distribution (e.g. permutation test; e.g. Winkler et al., 2014 Neuroimage), not against zero (the SD of the two distributions, if one is made of zeros, are critically different; moreover if values of standard - scrambled are all positive there is no point in testing it statistically against zero).

Thank you for this comment, we agree with the reviewer that for conceptual and methodological reasons it would be more appropriate to compare standard vs. scrambled rather than comparing the “standard - scrambled” subtraction against zero, also considering the second comment of the reviewer. Indeed, one of the strong point of this paradigm is that it allows to identify voice-selective responses without the need to perform an explicit subtraction as it is already present in the paradigm implicitly: for a selective response to voices to occur, the participants’ brain need to elicit a different responses to voices than to the other sounds, and there is no mandatory need to compare responses to different categories of sounds by subtracting brain responses to different categories of sounds.

Following the suggestion of the reviewer, the comparison performed in the revised manuscript is now “standard > scrambled” instead of “standard - scrambled > 0” (Materials and Methods, paragraph 2.1.7; Results, paragraph 3.1.2). The significance of the comparison and the associated statistical values will however not change as we are performing a paired sample t-test (data for the standard and scrambled conditions come from the same participants) and therefore the mathematical formula will be the same as for subtracting the two conditions and comparing their difference against zero.

As for the second point regarding the use of non-parametric tests (permutations) to asses statistical significance, we now have implemented a non-parametric procedure as follows. In each electrode (of the 128 electrodes), we performed the “standard > scrambled” comparison across participants using a paired t-test, and recorded our actual t-value from the t-test. Then we generated a null distribution of t-values by performing 10,000 permutations. In each permutation, we shuffled the values from the standard and the scrambled conditions (sum of the baseline subtracted amplitudes of the four significant harmonics) and recorded the null t-value. This resulted in a null distribution of 10,000 t-values. We then estimated the p-value as the probability of observing our actual t-value when compared with the generated null distribution. The p-values were corrected for multiple comparisons (n=128) using Bonferroni correction (this is a very conservative procedure needing p value to be below 0,0004 to be considered “significant”). Three out of the four electrodes that were identified with parametric testing were significant using non-parametric testing (see table and figure below; red lines in the graphs correspond to the t-values obtained with parametric testing). As parametric and non-parametric statistics led to very similar results, we would like to suggest to use parametric statistics for the “standard > scrambled” comparison for coherence with the rest of the text.

All p-values are Bonferroni corrected for the number of tests performed: 128, one per electrode.

Null distributions of t-values (10,000 permutations) of the four electrodes that reached significance using parametric statistics. Red lines in the graphs correspond to the t-values obtained with parametric testing.

2) In the manuscript it is often referred to the objectivity of the measure as it does not imply subtraction across conditions. However, the crucial comparison is standard - scrambled. This logical inconsistency leaves this reader rather puzzled.

The initial choice to include this extra comparison (standard- scrambled) was intended to stress the fact that the voice-selective response that we record here cannot be explained by low level acoustic of the voice alone: although this comparison is an extra step, we had included it as a proof of principle as we are presenting the paradigm for the first time. In the manuscript we are now stressing on the fact that this comparison is indeed considered as an extra step as FPAS does not require subtraction across conditions (Results, paragraph 3.1.2; Discussion). Moreover, notwithstanding the advantage of not requiring an explicit subtraction across conditions, the objectivity of FPAS mainly lies in the fact that the experimenter knows a priori the frequencies at which a voice-selective response is expected.

3) It is reported that following the scrambling procedure stimuli are not recognizable any more. Is there any behavioural data in support this claim? This would be of help to interpret what participants are perceiving.

We had indeed conducted a behavioral experiment to support this claim that was not included in the first submitted version of the manuscript. The experiment is now included in the text of the revised manuscript as experiment 3. Briefly, in the behavioral experiment participants listened to the sounds used to create the sequences of the EEG experiments (standard, scrambled and harmonic) that were presented either embedded in short sequences (sequence task, to mimic sound presentation as in the EEG experiments) or in isolation (isolation task). After each presentation, participants had to indicate whether they had heard a voice or not. Our results suggest that the frequency scrambling technique effectively disrupted the intelligibility of the sounds of the scrambled condition (see figure below; Materials and methods, paragraph 2.3; Results, paragraph 3.3).

Behavioral detection of voices (experiment 3 and figure 5 in the manuscript). Sensitivity indices for the sequence (left) and isolation (right) tasks, for the three conditions. The bar plots represent the group average of d’, each dot represents an individual participant’s d’ score.

4) More on the previous point, the first harmonic is significant even for the scrambled condition. This might reveal that participants were to some extents discriminating between base and target frequency even for the scrambled condition. The authors should provide deeper comments on this, especially if no measure of recognizability (see point 3) is available.

Good point. If it is true that the first harmonic for the scrambled condition is significant, it has to be noted that the elicited response is much lower than the one elicited by the standard condition (“[...] in regions where the response was peaking for the standard condition, the amplitude of the response to voice-scrambled stimuli was of 50.6% of the response to voices for the left ROI and only 18.8% for the right ROI", Figure 2E).

We think that this lower yet significant response might be due to two possible and not mutually exclusive explanations. First, this response might be due to the low-level acoustic features shared by voices and scrambled voices (i.e. frequency content): despite the fact that voice selective responses elicited by the standard condition could not be fully explained by low-level acoustic features alone, it might be the case that these features contribute in the response. In fact, feature dependence is not in conflict with a categorical coding hypothesis (Bracci et al., 2017), and preferential response biases observed in voice regions to specific acoustic features (Moerel et al., 2012, Lewis et al., 2009, Santoro et al., 2017) could serve as a scaffold or facilitate the emergence of a higher-level categorical responses to voices through experience. Second, although the scrambling procedure disrupted the intelligibility of the sounds (see our response to the point above), participants’ performance in categorizing scrambled sounds was still above chance, suggesting that some residual sound recognizability might have still been present (see figure 5). The scrambling procedure implemented in our study requires to choose two parameters: (i) the width of the frequency window and (ii) whether to impose the temporal envelope of the original sounds on the scrambled sounds or not. These parameters are chosen trying to find the best compromise between having scrambled sounds whose frequency spectra and temporal envelopes are as close as possible to the original sounds (to provide the best match of physical properties; see Figure 1C) while not being recognizable anymore (see figure 5). Using a wider frequency window and/or not imposing the temporal envelope of the original sounds could have disrupted sound intelligibility even more and possibly not elicit a response to the scrambled condition, but those stimuli would have been not as close to the original sounds for frequency content as the one used in our experiment. Further investigation would be needed to shed light into how these parameters affect sound intelligibility and voice-selective responses.

These considerations have now been included in the revised text (see Discussion).

5) At least for the group level, point of views of topo plots should be the same for base and target frequency (Figure 2 and 3) to appreciate how the topography changes.

Following this recommendation, we are now presenting the same points of view for the base and target frequencies at the group level for the two EEG experiments (see below).

Figure 2. Experiment 1 - Voice versus object sounds. A) Responses at the base (top) and at the target frequency (bottom) for the standard (left) and scrambled (right) conditions as topographic head plots. Topographic head plots were obtained by summing the baseline subtracted amplitude at the significant harmonics. B) Source localization analysis revealed stronger coherence values at the target frequency for the standard condition (standard > scrambled) over right superior and middle temporal areas (voxels with intensity above the 95th percentile are highlighted in red). C) Signal-to-noise ratio (SNR) of voice-selective responses obtained by averaging SNR values of ROIvoice electrodes as a function of number of stimulation sequences/minutes of recording for the standard condition. D) SNR averaged spectra of ROIvoice electrodes. E) Sum of the baseline corrected amplitude (considering 10 bins on each side, excluding the immediately adjacent ones) of voice selective responses at the four significant harmonics for the standard (blue) and scrambled condition (orange). F) Responses at the target frequency for the standard (blue) and scrambled (orange) conditions as the sum of baseline subtracted amplitude at four harmonics in the left and right ROIs. G) There is no correlation between responses at the left and right ROIs across the standard and scrambled conditions.

Figure 4. Experiment 2 - Voice versus musical instruments. A) Responses at the base and at the target frequency as topographic head plots. Topographic head plots were obtained by summing the baseline subtracted amplitude at the significant harmonics. B) SNR averaged spectra of ROIvoice electrodes.

6) Minor: the mixed use of epoch, trial and sequences is confounding.

In the revised manuscript we are now referring to “stimulation sequence” or “sequence", while the term “epoch” is only used in the data analysis section. The term “trial” is now used in the case of the behavioral experiment (experiment 3) that has been added in the manuscript following previous comment #3).

## References

[B1] Agus TR, Paquette S, Suied C, Pressnitzer D, Belin P (2017) Voice selectivity in the temporal voice area despite matched low- level acoustic cues. Sci Rep 7:11526. 10.1038/s41598-017-11684-1 28912437 PMC5599610

[B2] Akalin Acar Z, Makeig S (2013) Effects of forward model errors on EEG source localization. Brain Topogr 26:378–396. 10.1007/s10548-012-0274-6 23355112 PMC3683142

[B3] Alonso-Prieto E, Van Belle G, Liu-Shuang J, Norcia AM, Rossion B (2013) The 6Hz fundamental stimulation frequency rate for individual face discrimination in the right occipito-temporal cortex. Neuropsychologia 51:2863–2875. 10.1016/j.neuropsychologia.2013.08.018 24007879

[B4] Belin P, Zatorre RJ, Lafaille P, Ahad P, Pike B (2000) Voice-selective areas in human auditory cortex. Nature 403:309–312. 10.1038/35002078 10659849

[B5] Belin P, Zatorre RJ, Ahad P (2002) Human temporal-lobe response to vocal sounds. Brain Res Cogn Brain Res 13:17–26. 10.1016/S0926-6410(01)00084-2 11867247

[B6] Belin P, Fecteau S, Bédard C (2004) Thinking the voice: neural correlates of voice perception. Trends Cogn Sci 8:129–135. 10.1016/j.tics.2004.01.008 15301753

[B7] Belin P, Fillion-Bilodeau S, Gosselin F (2008) The Montreal affective voices: a validated set of nonverbal affect bursts for research on auditory affective processing. Behav Res Methods 40:531–539. 10.3758/BRM.40.2.531 18522064

[B8] Belin P, Bestelmeyer PEG, Latinus M, Watson R (2011) Understanding voice perception. Br J Psychol 102:711–725. 10.1111/j.2044-8295.2011.02041.x 21988380

[B91] Boersma P (2001) Praat, a system for doing phonetics by computer. Glot International 5:341–345.

[B9] Bottari D, Bednaya E, Dormal G, Villwock A, Dzhelyova M, Grin K, Pietrini P, Ricciardi E, Rossion B, Röder B (2020) EEG frequency-tagging demonstrates increased left hemispheric involvement and crossmodal plasticity for face processing in congenitally deaf signers. Neuroimage 223:117315. 10.1016/j.neuroimage.2020.117315 32882385

[B10] Bracci S, Ritchie JB, Op De Beeck H (2017) On the partnership between neural representations of object categories and visual features in the ventral visual pathway. Neuropsychologia 105:153–164. 10.1016/j.neuropsychologia.2017.06.010 28619529 PMC5680697

[B11] Brainard DH (1997) The psychophysics toolbox. Spat Vis 10:433–436. 10.1163/156856897X00357 9176952

[B12] Charest I, Pernet CR, Rousselet GA, Quiñones I, Latinus M, Fillion-Bilodeau S, Chartrand J, Belin P (2009) Electrophysiological evidence for an early processing of human voices. BMC Neurosci 10:127. 10.1186/1471-2202-10-127 19843323 PMC2770575

[B13] De Lucia M, Clarke S, Murray MM (2010) A temporal hierarchy for conspecific vocalization discrimination in humans. J Neurosci 30:11210–11221. 10.1523/JNEUROSCI.2239-10.2010 20720129 PMC6633490

[B14] Ding J, Sperling G, Srinivasan R (2006) Attentional modulation of SSVEP power depends on the network tagged by the flicker frequency. Cereb Cortex 16:1016–1029. 16221931 10.1093/cercor/bhj044 PMC1880883

[B15] Dormal G, Pelland M, Rezk M, Yakobov E, Lepore F, Collignon O (2018) Functional preference for object sounds and voices in the brain of early blind and sighted individuals. J Cogn Neurosci 30:86–106. 28891782 10.1162/jocn_a_01186

[B16] Frühholz S, Belin P (2018) The Oxford handbook of voice perception. Oxford: Oxford University Press.

[B17] Giordano BL, McAdams S, Zatorre RJ, Kriegeskorte N, Belin P (2013) Abstract encoding of auditory objects in cortical activity patterns. Cereb Cortex 23:2025–2037. 10.1093/cercor/bhs162 22802575

[B18] Goto M, Hashiguchi H, Nishimura T, Oka R (2003) RWC music database: music genre database and musical instrument sound database. Proceedings of the International Conference on Music Information Retrieval (ISMIR).

[B19] Gross J, Kujala J, Hämäläinen M, Timmermann L, Schnitzler A, Salmelin R (2001) Dynamic imaging of coherent sources: studying neural interactions in the human brain. Proc Natl Acad Sci USA 98:694–699. 10.1073/pnas.98.2.694 11209067 PMC14650

[B20] Halder T, Talwar S, Jaiswal AK, Banerjee A (2019) Quantitative evaluation in estimating sources underlying brain oscillations using current source density methods and beamformer approaches. eNeuro 6:ENEURO.0170-19.2019–14. 10.1523/ENEURO.0170-19.2019 PMC6709228 31311804

[B21] Kleiner M, Brainard D, Pelli D, Ingling A, Murray R, Broussard C (2007) What’s new in Psychtoolbox-3? Perception. 36(ECVP Abstract Supplement):14. Whats New 36:14.

[B22] Leaver AM, Rauschecker JP (2010) Cortical representation of natural complex sounds: effects of acoustic features and auditory object category. J Neurosci 30:7604–7612. 10.1523/JNEUROSCI.0296-10.2010 20519535 PMC2930617

[B23] Levy DA, Granot R, Bentin S (2003) Neural sensitivity to human voices: ERP evidence of task and attentional influences. Psychophysiology 40:291–305. 10.1111/1469-8986.00031 12820870

[B24] Lewis JW, Talkington WJ, Walker NA, Spirou GA, Jajosky A, Frum C, Brefczynski-Lewis JA (2009) Human cortical organization for processing vocalizations indicates representation of harmonic structure as a signal attribute. J Neurosci 29:2283–2296. 10.1523/JNEUROSCI.4145-08.2009 19228981 PMC2774090

[B25] Liu-Shuang J, Torfs K, Rossion B (2016) An objective electrophysiological marker of face individualisation impairment in acquired prosopagnosia with fast periodic visual stimulation. Neuropsychologia 83:100–113. 10.1016/j.neuropsychologia.2015.08.023 26318239

[B26] Lochy A, Van Belle G, Rossion B (2015) A robust index of lexical representation in the left occipito-temporal cortex as evidenced by EEG responses to fast periodic visual stimulation. Neuropsychologia 66:18–31. 10.1016/j.neuropsychologia.2014.11.007 25448857

[B27] Luck SJ (2014) An Introduction to the event-related potential technique, Ed 2. Cambridge: The MIT Press.

[B28] Macmillan NA, Creelman CD (2004) Detection theory: a user’s guide, Ed 2. Mahwah: Lawrence Erlbaum Associates.

[B29] Moerel M, De Martino F, Formisano E (2012) Processing of natural sounds in human auditory cortex: tonotopy, spectral tuning, and relation to voice sensitivity. J Neurosci 32:14205–14216. 10.1523/JNEUROSCI.1388-12.2012 23055490 PMC6622378

[B30] Murray MM, Camen C, Gonzalez AS, Bovet P, Clarke S (2006) Rapid brain discrimination of sounds of objects. J Neurosci 26:1293–1302. 10.1523/JNEUROSCI.4511-05.2006 16436617 PMC6674563

[B31] Norcia AM, Appelbaum LG, Ales JM, Cottereau BR, Rossion B (2015) The steady-state visual evoked potential in vision research: a review. J Vis 15:4. 10.1167/15.6.4 PMC4581566 26024451

[B32] Norman-Haignere SV, McDermott JH (2018) Neural responses to natural and model-matched stimuli reveal distinct computations in primary and nonprimary auditory cortex. PLoS Biol 16:e2005127. 10.1371/journal.pbio.2005127 30507943 PMC6292651

[B33] Ogg M, Carlson TA, Slevc LR (2020) The rapid emergence of auditory object representations in cortex reflect central acoustic attributes. J Cogn Neurosci 32:111–123. 10.1162/jocn_a_01472 31560265

[B34] Oostenveld R, Fries P, Maris E, Schoffelen JM (2011) FieldTrip: open source software for advanced analysis of MEG, EEG, and invasive electrophysiological data. Comput Intell Neurosci 2011:1–9. 10.1155/2011/156869 21253357 PMC3021840

[B35] Patterson RD, Uppenkamp S, Johnsrude IS, Griffiths TD (2002) The processing of temporal pitch and melody information in auditory cortex. Neuron 36:767–776. 10.1016/S0896-6273(02)01060-7 12441063

[B36] Peelen MV, Downing PE (2017) Category selectivity in human visual cortex: beyond visual object recognition. Neuropsychologia 105:177–183. 10.1016/j.neuropsychologia.2017.03.033 28377161

[B37] Pelli DG (1997) The VideoToolbox software for visual psychophysics: transforming numbers into movies. Spat Vis 10:437–442. 10.1163/156856897X00366 9176953

[B38] Popov T, Oostenveld R, Schoffelen JM (2018) FieldTrip made easy: an analysis protocol for group analysis of the auditory steady state brain response in time, frequency, and space. Front Neurosci 12:1–11. 30356712 10.3389/fnins.2018.00711 PMC6189392

[B39] Regan D (1966) Some characteristics of average steady-state and transient responses evoked by modulated light. Electroencephalogr Clin Neurophysiol 20:238–248. 4160391 10.1016/0013-4694(66)90088-5

[B92] Regan D (1989) Human brain electrophysiology: evoked potentials and evoked magnetic fields in science and medicine. Elsevier, Amsterdam.

[B40] Retter TL, Rossion B (2016) Uncovering the neural magnitude and spatio-temporal dynamics of natural image categorization in a fast visual stream. Neuropsychologia 91:9–28. 10.1016/j.neuropsychologia.2016.07.028 27461075

[B41] Retter TL, Jiang F, Webster MA, Rossion B (2018) Dissociable effects of inter-stimulus interval and presentation duration on rapid face categorization. Vision Res 145:11–20. 10.1016/j.visres.2018.02.009 29581059 PMC6485415

[B42] Retter TL, Jiang F, Webster MA, Rossion B (2020) All-or-none face categorization in the human brain. Neuroimage 213:116685. 10.1016/j.neuroimage.2020.116685 32119982 PMC7339021

[B43] Rice GE, Watson DM, Hartley T, Andrews TJ (2014) Low-level image properties of visual objects predict patterns of neural response across category-selective regions of the ventral visual pathway. J Neurosci 34:8837–8844. 10.1523/JNEUROSCI.5265-13.2014 24966383 PMC4069357

[B44] Rossion B, Torfs K, Jacques C, Liu-Shuang J (2015) Fast periodic presentation of natural images reveals a robust face-selective electrophysiological response in the human brain. J Vis 15:18. 10.1167/15.1.18 25597037

[B93] RStudio Team (2018) RStudio: Integrated development for R. RStudio, Inc., Boston, MA. Available at http://www.rstudio.com/.

[B45] Santoro R, Moerel M, De Martino F, Valente G, Ugurbil K, Yacoub E (2017) Reconstructing the spectrotemporal modulations of real-life sounds from fMRI response patterns. Proc Natl Acad Sci USA 114:4799–4804. 28420788 10.1073/pnas.1617622114 PMC5422795

[B46] Staeren N, Renvall H, De Martino F, Goebel R, Formisano E (2009) Sound categories are represented as distributed patterns in the human auditory cortex. Curr Biol 19:498–502. 10.1016/j.cub.2009.01.066 19268594

[B47] Tanner WP, Swets JA (1954) A decision-making theory of visual detection. Psychol Rev 61:401–409. 10.1037/h0058700 13215690

[B48] Von Kriegstein K, Eger E, Kleinschmidt A, Giraud AL (2003) Modulation of neural responses to speech by directing attention to voices or verbal content. Brain Res Cogn Brain Res 17:48–55. 10.1016/S0926-6410(03)00079-X 12763191

[B49] Warren JD, Jennings AR, Griffiths TD (2005) Analysis of the spectral envelope of sounds by the human brain. Neuroimage 24:1052–1057. 10.1016/j.neuroimage.2004.10.031 15670682

[B50] Winkler AM, Ridgway GR, Webster MA, Smith SM, Nichols TE (2014) Permutation inference for the general linear model. Neuroimage 92:381–397. 10.1016/j.neuroimage.2014.01.060 24530839 PMC4010955

